# Robust 2D Otsu's Algorithm for Uneven Illumination Image Segmentation

**DOI:** 10.1155/2020/5047976

**Published:** 2020-08-11

**Authors:** Jiangwa Xing, Pei Yang, Letu Qingge

**Affiliations:** ^1^Research Center of Basic Medical Sciences, Medical College, Qinghai University, Xining 810016, China; ^2^Department of Computer Technology and Application, Qinghai University, Xining 810016, China; ^3^Department of Computer Science, North Carolina A&T State University, Greensboro, NC 27411, USA

## Abstract

Otsu's algorithm is one of the most well-known methods for automatic image thresholding. 2D Otsu's method is more robust compared to 1D Otsu's method. However, it still has limitations on salt-and-pepper noise corrupted images and uneven illumination images. To alleviate these limitations and improve the overall performance, here we propose an improved 2D Otsu's algorithm to increase the robustness to salt-and-pepper noise together with an adaptive energy based image partition technology for uneven illumination image segmentation. Based on the partition method, two schemes for automatic thresholding are adopted to find the best segmentation result. Experiments are conducted on both synthetic and real world uneven illumination images as well as real world regular illumination cell images. Original 2D Otsu's method, MAOTSU_2D, and two latest 1D Otsu's methods (Cao's method and DVE) are included for comparisons. Both qualitative and quantitative evaluations are introduced to verify the effectiveness of the proposed method. Results show that the proposed method is more robust to salt-and-pepper noise and acquires better segmentation results on uneven illumination images in general without compromising its performance on regular illumination images. For a test group of seven real world uneven illumination images, the proposed method could lower the ME value by 15% and increase the DSC value by 10%.

## 1. Introduction

As a fundamental technique for computer vision related applications, image segmentation has been studied for decades [1–6]. Despite the development of many complex segmentation algorithms including deep learning-based methods, automatic thresholding is still widely adopted and keeps evolving due to its simplicity and effectiveness [4, 5]. To date, numerous automatic thresholding algorithms have been proposed [5], which can be further categorized into either local thresholding or global thresholding. Local thresholding tries to seek multiple threshold values based on localized gray level information, while global thresholding calculates the threshold value only using global information to make it simpler and more efficient. There are lots of adaptive thresholding methods such as Otsu's method, Kapur's method, and entropy based method [5]. Otsu's method is one of the most well-known and effective global thresholding algorithms proposed by Otsu in 1979 [6]. It is so far still widely used in many applications including document image binarization [7], medical image processing [8], life science [9], and combating infectious diseases such as coronavirus disease (COVID-19) [10]. However, it still suffers some disadvantages and fails in certain cases for optimal image segmentation.

To improve the performance of Otsu's method, many scholars have done significant amount of work to study its characteristics and improve the thresholding algorithm since it was proposed. Many one-dimensional (1D) improved Otsu's algorithms are firstly developed. Xu et al. studied the characteristics of the threshold value acquired using Otsu's method and came to the conclusion that the threshold biases toward the class with larger variance when the intraclass variances of two classes are different [11]. Based on this characteristic, Xu proposed an improved Otsu's method by constraining the search range of gray levels. In order to make the threshold value more likely located at the valley of image histogram, Ng suggested adopting gray level probability as a valley metric and proposed the valley emphasis (VE) method [12]. A penalty factor using the proposed valley metric was introduced into the object function, which was proved to be effective on defect detection issue. Later, Fan pointed out that in Ng's method only the valley point value of the histogram was weighted, and it could be beneficial to use the neighborhood information as well [13]. Based on this conception, Fan developed the neighborhood valley emphasis (NVE) method which adds the neighborhood information by smoothing the histogram using an average filter. Furthermore, we have previously proposed a new modified valley emphasis metric using second order derivative (DVE) to better fit the real valley of histogram [14]. In addition, there are many other 1D improved Otsu's algorithms [3, 15, 16]. However, most of these algorithms could not deal with images corrupted by noise.

In order to overcome the disadvantage shared by most 1D Otsu's algorithms, Liu et al. proposed to extend Otsu's method to a 2D histogram. The 2D Otsu's method utilizes both the pixel's gray level and the average gray level of its neighborhood and is experimentally proved to perform better than 1D Otsu's method on images corrupted by noise [17]. However, it still has some limitations such as computationally expensive, poor robustness to salt-and-pepper noise. To decrease computation complexity, Gong et al. proposed a fast recursive algorithm which can reduce both the computation complexity and required memory space compared to the original 2D Otsu's method [18]. Later, Yue et al. decomposed the 2D Otsu's method into two 1D Otsu's algorithms and calculated the optimal threshold of each 1D Otsu's algorithm independently [19]. Compared to the original 2D Otsu's method, the computational complexity of Yue's method is reduced to O(*L*). Another representative way to reduce the consuming time is to employ heuristic and metaheuristic algorithms in 2D Otsu's and Otsu based multilevel image thresholding [20, 21]. Furthermore, to alleviate 2D Otsu's limitation of lack of robustness to salt-and-pepper noise, Sha et al. proposed a robust 2D Otsu's method called MAOTSU_2D [22]. In Sha's work, median filtering followed by average filtering is adopted to build the 2D histogram instead of only using average filtering, and a region postprocessing step is proposed to deal with pixels of noise and edges. Other improved 2D Otsu's algorithms such as curve thresholding segmentation method [23] and grid box filter based 2D Otsu's method [24] have also been proposed. However, these existing algorithms usually generate poor segmentation results on uneven illumination images and salt-and-pepper corrupted images, and these limitations of 2D Otsu's method will be discussed in more detail in the next section.

Due to the stability of environment light, the object size, and other reasons, uneven illumination is a common problem in image capturing [25, 26], and there exist some typical technologies for uneven lighting image enhancement and segmentation. One representative category is to use local threshold value for pixel or subregions. Parker [27] proposed a pixel level thresholding using local intensity gradient (LIG) and a region growing strategy for badly illuminated images. In [26], an adaptive window selection method based on Lorentz information measure has been studied, and the adaptive selected windows (regions) were thresholded by Otsu's method. Pradhan et al. proposed two adaptive thresholding schemes for uneven illuminated images. One is a window merging method based on Lorentz information measure, and the other is a window growing technology using the notion of entropy. Based on the above two strategies, the whole image was divided into some fixed subregions, and Otsu's method is then applied for each region [28]. As the initial size of window can significantly affect the segmentation accuracy, Saini and Dutta proposed a method to dynamically determine the initial size of the window using the parameters of the input image and addressed the uneven illumination image segmentation problem based on adaptive windowing technology and incremental window growing method [29]. Besides the above introduced pixel level or portioning algorithms, image correction technology is another effective solution. In [30], Ashour et al. studied the low contrast tomography image enhancement problem using log transform, and Cuckoo search (CS) algorithm was adopted to determine the optimal parameter setting for log transform. In Yu's work [31], a wave transformation method is proposed for uneven lighting images. The input image was regarded as a gray wave, and relative characteristic of each pixel was computed, and nonlocal adaptive spatial constraints and edge information were introduced for wave transformation. After that, intuitionistic fuzzy set was implemented on the transformed image for segmentation. In addition, there are many other technologies for uneven image enhancement and segmentation including machine learning method [32] and background correction [33].

As introduced above, the existing uneven methods usually not directly improve the original segmentation algorithm. For better overcoming the disadvantages of 2D Otsu's algorithm, in this paper, we are focusing on developing an improved 2D Otsu's method to generate better segmentation performance on both uneven illumination images and salt-and-pepper corrupted images. The main contributions of this paper are as follows: (1) firstly, a novel 2D histogram construction strategy based on median and average filters is proposed to enhance the algorithm's robustness to salt-and-pepper noise. (2) Secondly, an energy based partitioning technology is introduced to find the best splitting line for uneven illumination images. (3) Lastly, two schemes based on the new 2D histogram construction strategy and partitioning method are proposed, and their robustness to salt-and-pepper noise is studied and the segmentation performance is evaluated both qualitatively and quantitatively on uneven illumination testing images and on an extra cell dataset. The remainder of this paper is organized as follows. In Section 2, we review the original 2D Otsu's method briefly, followed by introducing the proposed 2D histogram construction strategy and partition technology based 2D Otsu's methods in Section 3.  Section 4 presents the experimental results and analysis on both synthetic and numerous real world images, and Section 5 gives the concluding remarks.

## 2. Two-Dimensional Otsu's Method

### 2.1. Review of 2D Otsu's Method

In this section we will briefly review the traditional 2D Otsu's method firstly. Suppose *I* is an image represented in *L* gray levels with *m* rows and *n* columns, and *G* is the corresponding averaged image; then pixel gray level in *G* can be defined as follows:

(1) $$\begin{array}{c} G\left(x,y\right)=\sum_{\tilde{x}=x-\left(k-1\right)/2}^{x+\left(k-1\right)/2}\sum_{\tilde{y}=y-\left(k-1\right)/2}^{y+\left(k-1\right)/2}I\left(\tilde{x},\tilde{y}\right), \end{array}$$

where *I*(*x*, *y*) and *G*(*x*, *y*) represent the gray level of pixel at (*x*, *y*) in *I* and *G*, respectively. *k* represents the size of the filter, and the value of *k* is set to be 3 in this paper. Let *i*, *j* be pixel gray level of original image and averaged image; then (*i*, *j*) is a gray level pair representing that the pixel gray level in image *I* is *i*, and the gray level of the corresponding pixel at the same location in the averaged image *G* is *j*. Suppose *f*_*ij*_ is the pixel number of (*i*, *j*), then the two-dimensional probability density function can be defined as

(2) $$\begin{array}{c} p_{ij}=\frac{f_{ij}}{m\times n}, \end{array}$$

where *i*, *j* ∈ [0, *L* − 1] and ∑_i_∑_j_*p*_*ij*_=1. The average vector of the 2D histogram is as follows:

(3) $$\begin{array}{c} u_{T}={\left(u_{Ti},u_{Tj}\right)}^{T}={\left(\sum_{i=0}^{L-1}\sum_{j=0}^{L-1}ip_{ij},\sum_{i=0}^{L-1}\sum_{j=0}^{L-1}jp_{ij}\right)}^{T}. \end{array}$$

**Figure 1:**
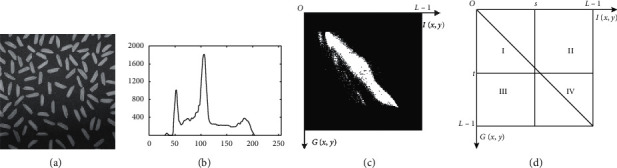
Image rice and its histogram, 2D histogram, and corresponding division. (a) Original image. (b) Histogram. (c) Projection of 2D histogram. (d) Corresponding regional division of 2D histogram.

Figure 1 shows the histogram and projection of 2D histogram of image rice.  Figure 1(b) is the one-dimensional histogram and Figure 1(c) is the projection of 2D histogram of a gray scale image. The relationship between Figure 1(b) and Figure 1(c) is that pixel number with gray level *l* in Figure 1(b) can be obtained by accumulating the pixel intensity of the *l*th column in Figure 1(c). And for a given threshold pair (*s*, *t*), the 2D histogram can be split into four parts marked as I, II, III, and IV by line *I*(*x*, *y*)=*s* and *G*(*x*, *y*)=*t* as shown in Figure 1(d). Gray level of corresponding pixels in background or foreground should be approximate as these areas are relatively smooth, and this results in the fact that regions I and IV around the diagonal are corresponding to the background and foreground, respectively. In practice, regions II and III are negligible as they correspond to noise pixels and edges. Finally in 2D Otsu's method, for a given threshold pair (*s*, *t*), pixels can be partitioned into two sets, *C*_0_ and *C*_1_ (background and foreground), and the class occurrence probabilities can be expressed as

(4) $$\begin{array}{c} \omega_{0}=P\left(C_{0}\right)=\sum_{i=0}^{s}\sum_{j=0}^{t}p_{ij},\\ \omega_{1}=P\left(C_{1}\right)=\sum_{i=s+1}^{L-1}\sum_{j=t+1}^{L-1}p_{ij}. \end{array}$$

The corresponding mean vectors of *C*_0_ and *C*_1_ are

(5) $$\begin{array}{c} u_{0}={\left(u_{0i},u_{0j}\right)}^{T}={\left(\sum_{i=0}^{s}\sum_{j=0}^{t}\frac{ip_{ij}}{\omega_{0}},\sum_{i=0}^{s}\sum_{j=0}^{t}\frac{jp_{ij}}{\omega_{0}}\right)}^{T},\\ u_{1}={\left(u_{1i},u_{1j}\right)}^{T}={\left(\sum_{i=s+1}^{L-1}\sum_{j=t+1}^{L-1}\frac{ip_{ij}}{\omega_{1}},\sum_{i=s+1}^{L-1}\sum_{j=t+1}^{L-1}\frac{jp_{ij}}{\omega_{1}}\right)}^{T}. \end{array}$$

Due to the assumption that occurrence of image data away from the diagonal of 2D histogram is negligible, we can get the following approximate expression:

(6) $$\begin{array}{c} \omega_{0}+\omega_{1}≃1,u_{T}=\omega_{0}u_{0}+\omega_{1}u_{1}. \end{array}$$

The between-class variance in 2D Otsu's algorithm can be then defined as

(7) $$\begin{array}{c} tr\left(\sigma_{B}\right)=tr\left(\sum_{k=0}^{1}\omega_{k}\left[\left(u_{k}-u_{T}\right){\left(u_{k}-u_{T}\right)}^{T}\right]\right). \end{array}$$

Then, we can get the optimal threshold pair (*s*^*∗*^, *t*^*∗*^) by maximizing the between-class variance defined in formula (7):

(8) $$\begin{array}{c} \left(s^{*},t^{*}\right)=\underset{0<s<L,0<t<L}{\arg\max}\left(tr\left(\sigma_{B}\right)\right). \end{array}$$

### 2.2. Limitation Analysis of 2D Otsu's Method

**Figure 2:**
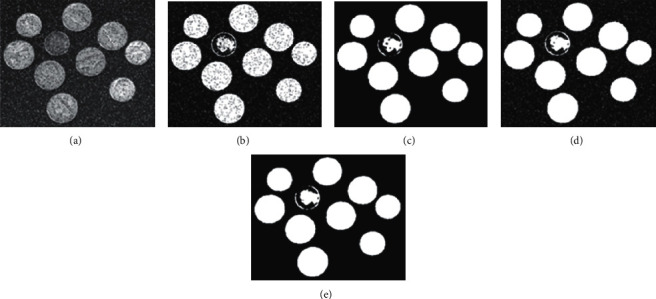
Segmentation results on salt-and-pepper corrupted image. (a) Image coins corrupted by salt-and-pepper noise (*δ* = 0.1). Segmentation results of (b) traditional 2D Otsu's method, (c) MAOTSU_2D, (d) MAOTSU_2D without postprocess, and (e) our proposed scheme 2.

While 2D Otsu's algorithm is more robust to noises than 1D Otsu's method, it still has limitations on salt-and-pepper noise corrupted images as well as uneven illumination images.  Figure 2 demonstrates the segmentation results using 2D Otsu's method, MAOTSU_2D, and our proposed algorithm on image coins corrupted by salt-and-pepper noise.  Figure 2(b) indicates that the traditional 2D Otsu's method could not deal with salt-and-pepper noise well. In order to obtain the robustness to salt-and-pepper noise, a median-average filter was adopted in MAOTSU_2D.  Figure 2(c) shows the result of MAOTSU_2D on Figure 2(a), and the method produces a better segmentation result. However, it is worth noting that a median-average filter and a postprocess strategy were both adopted in MAOTSU_2D.  Figure 2(d) shows the result of MAOTSU_2D without the postprocess step, and it is obvious that the noise corrupted pixels in background area were not well processed. In this paper, we introduce an improved 2D histogram constructing strategy for better dealing with salt-and-pepper noise, and Figure 2(e) is the segmentation result of our proposed method.

**Figure 3:**
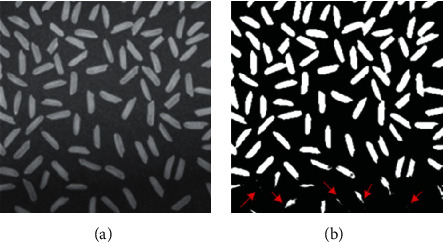
Segmentation results on an uneven illumination image. (a) Image rice. (b) Segmentation result of traditional 2D Otsu's method.

In addition to salt-and-pepper noise, traditional 2D Otsu's method usually produces poor segmentation on uneven illumination images.  Figure 3 shows the segmentation result using traditional 2D Otsu's method on an uneven illumination image.  Figure 3(a) is the typical image rice widely used in image processing field, and one remarkable characteristic of image rice is nonuniform illumination.  Figure 3(b) shows the segmentation result of traditional 2D Otsu's method, and the result is seriously affected by the uneven illumination. Pixels of both background and foreground of the bottom area are much darker than those of other regions, and the uneven illumination consequently results in a significant loss of foreground objects at the bottom area of the image pointed out by red arrows.

To solve the abovementioned problems of 2D Otsu's algorithm, in this paper, we focus on 2D histogram constructing to enhance the robustness of 2D Otsu's method to salt-and-pepper noise, and the image partitioning technology is studied to improve the algorithm's effectiveness on uneven illumination images.

## 3. Proposed Robust 2D Otsu's Algorithm

### 3.1. Overview of the Proposed Method

**Figure 4:**
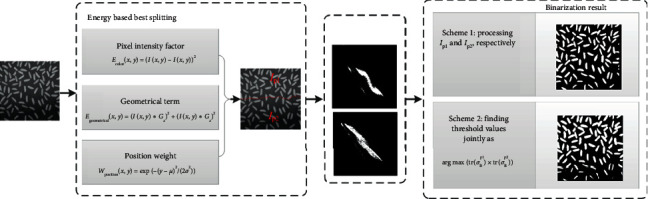
Block diagram of the proposed robust Otsu's algorithm.

The workflow of our proposed method is shown in Figure 4. Firstly, to alleviate the effect of uneven illumination, the test image would be split into two subimages with relatively uniform illumination using energy based best splitting method called splitting line. Next, an improved 2D Otsu's method named MMAOTSU_2D would be applied to construct the histograms of these two subimages. This improved 2D Otsu's method based on median and average filters is supposed to be effective to salt-and-pepper noise. Finally, two schemes of autothresholding methods are implemented to find the best segmentation result of the test image. Scheme 1 is to process the two subimages separately and find individual best threshold vector for each of the two subimages, and scheme 2 is to find the optimal threshold pair for the two parts jointly by solving the optimization problem. The final segmentation result would be the better one from the two schemes.

### 3.2. Two-Dimensional Histogram Constructing Strategy

As discussed in Section 2, the 2D histogram used in original 2D Otsu's method and MAOTSU_2D lacks robustness to salt-and-pepper noise, and in this section we will firstly discuss the strategy for constructing a robust 2D histogram. Motivated by the fact that median filter is effective to salt-and-pepper noise, we introduce a median filtering step in 2D histogram construction, and we name the 2D Otsu's method using the improved 2D histogram MMAOTSU_2D. The details of our proposed 2D histogram are as follows.

Firstly, we apply median filtering with *k* × *k* convolutional kernel on the original image *I* and obtain the median image *M* as

(9) $$\begin{array}{c} M\left(x,y\right)=\text{med}\left\{\left.I\left(\tilde{x},\tilde{y}\right)\right|x-\frac{k-1}{2}\leq \tilde{x}\leq x+\frac{k-1}{2},y-\frac{k-1}{2}\leq \tilde{y}\leq y+\frac{k-1}{2}\right\}. \end{array}$$

After we get the median image *M*, we calculate the average image as follows:

(10) $$\begin{array}{c} G\left(x,y\right)=\sum_{\tilde{x}=x-\left(k-1\right)/2}^{x+\left(k-1\right)/2}\sum_{\tilde{y}=y-\left(k-1\right)/2}^{y+\left(k-1\right)/2}M\left(\tilde{x},\tilde{y}\right). \end{array}$$

Finally, we construct the 2D histogram using median image *M* and median-average image *G*, and our strategy is different from that of original 2D Otsu's method and MAOTSU_2D.

**Figure 5:**
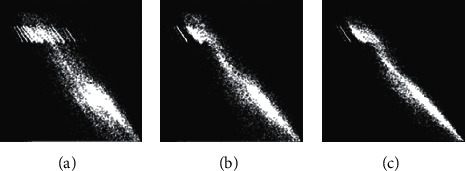
Projection of 2D histograms on image coins corrupted by salt-and-pepper noise (*δ*=0.1) using (a) original image and average image (original 2D Otsu's method), (b) original image and median-average image (MAOTSU_2D), and (c) median image and median-average image (the proposed).

Figure 5 is an intuitive presentation of 2D histograms of the original 2D Otsu's method (Figure 5(a)), MAOTST_2D algorithm (Figure 5(b)), and our proposed (Figure 5(c)). As introduced in Section 2, the 2D histogram can be partitioned into four regions. While the regions along the diagonal (regions I and IV in Figure 1(b)) correspond to the background and foreground, respectively, pixels in regions away from diagonal line represent noise and edges. Based on the above discussion, we can come to the conclusion that the more compact the 2D histogram is along the diagonal line, the less the influence of noise will be. As shown in Figure 5(a), the 2D histogram constructed using original image and average image is less compact along the diagonal line, and it is better when using the original image and median-average image (Figure 5(b)). Figure 5(c) shows the result of the proposed 2D histogram constructing strategy using median image and median-average image, and the 2D histogram is the most compact one among all the three 2D histograms. We will further discuss the effectiveness of the proposed 2D histogram constructing strategy later in the experimental section.

### 3.3. Partition Technology for Uneven Illumination Image Segmentation

Due to the limitation of traditional 2D Otsu's method for uneven illumination images, in this section we explore partition strategy to improve the algorithm's segmentation ability. It is a very straightforward idea to divide the uneven illumination images into some patches in which the illumination is uniform. However, how to divide the uneven illumination image adaptively is the key problem to be solved. Seam-line technology is widely used in image stitching [34–36], and an ideal seam-line makes the difference of both intensity and geometrical structure around the stitch line minimal [36]. Inspired by the success of seam-line technology in image mosaic, in this paper, we focus on dividing the uneven illumination image using a seam-line-like technology named splitting line. Unlike the seam-line, the splitting line makes the intensity difference around it maximum, and it should avoid crossing geometrical structure as much as possible as well. Consequently, we can define the energy function as

(11) $$\begin{array}{c} E\left(x,y\right)=\omega_{1}\cdot E_{\text{color}}\left(x,y\right)-\omega_{2}\cdot E_{\text{geometrical}}\left(x,y\right), \end{array}$$

where *ω*_1_ and *ω*_2_ are weight parameters, and in practice we set the values of *ω*_1_ and *ω*_2_ as $\sqrt{2}$ and 1, respectively. In this paper, we assume that the illumination is changing vertically, and we just partition the image into two parts. The *E*_color_(*x*, *y*) term in formula (11) reflects the pixel intensity change, and it is defined as

(12) $$\begin{array}{c} E_{\text{color}}\left(x,y\right)={\left(I\left(x,y\right)-I\left(x,y-1\right)\right)}^{2}. \end{array}$$

In order to compute the color energy for the first row, we add image padding using the first row of the image in our implementation.

The other term *E*_geometrical_(*x*, *y*) is a penalty factor, and it makes the splitting line avoid crossing texture rich area as much as possible. The magnitude of gradient is adopted to represent the *E*_geometrical_(*x*, *y*) term, and we use Sobel operator to calculate the gradient. The final form of *E*_geometrical_(*x*, *y*) is shown in the following formula:

(13) $$\begin{array}{c} E_{\mathrm{geometrical}}\left(x,y\right)={\left(I\left(x,y\right)*G_{x}\right)}^{2}+{\left(I\left(x,y\right)*G_{y}\right)}^{2}, \end{array}$$

where *G*_*x*_ and *G*_*y*_ represent the directional derivative templates as follows:

(14) $$\begin{array}{c} G_{x}=\left[\begin{array}{ccc} -1 & 0 & 1\\ -2 & 0 & 2\\ -1 & 0 & 1 \end{array}\right],\\ G_{y}=\left[\begin{array}{ccc} -1 & -2 & -1\\ 0 & 0 & 0\\ 1 & 2 & 1 \end{array}\right]. \end{array}$$

In addition to the above discussed requirements for the splitting line, we add position weight to the energy function to make the divided two parts as even as possible. The position weight is defined using a Gaussian function defined below:

(15) $$\begin{array}{c} W_{\text{position}}\left(x,y\right)=\exp\left(-\frac{{\left(y-\mu \right)}^{2}}{2\sigma^{2}}\right) \end{array}$$

For an image of size *m* × *n*, parameter *μ* in formula (15) is set to be *m*/2 due to the effect of position weight, and *σ* is empirically set to $\sqrt{m/2}$ in our implementation. Then, the position weight can be represented as

(16) $$\begin{array}{c} W_{\text{position}}\left(x,y\right)=\exp\left(-\frac{{\left(y-\left(m/2\right)\right)}^{2}}{m}\right). \end{array}$$

The energy function is finally changed to formula (17) after adding the position weight:

(17) $$\begin{array}{c} E\left(x,y\right)=W_{\text{position}}\left(x,y\right)\cdot \left(\omega_{1}\cdot E_{\text{color}}\left(x,y\right)-\omega_{2}\cdot E_{\text{geometrical}}\left(x,y\right)\right)\\ =\exp\left(-\frac{{\left(y-\left(m/2\right)\right)}^{2}}{m}\right)\cdot \left(\sqrt{2}\cdot E_{\text{color}}\left(x,y\right)-E_{\text{geometrical}}\left(x,y\right)\right)\\ =\sqrt{2}\cdot \exp\left(-\frac{{\left(y-\left(m/2\right)\right)}^{2}}{m}\right)\cdot {\left(I\left(x,y\right)-I\left(x,y-1\right)\right)}^{2}\\ -\exp\left(-\frac{{\left(y-\left(m/2\right)\right)}^{2}}{m}\right)\cdot \left({\left(I\left(x,y\right)*G_{x}\right)}^{2}+{\left(I\left(x,y\right)*G_{y}\right)}^{2}\right). \end{array}$$

**Figure 6:**
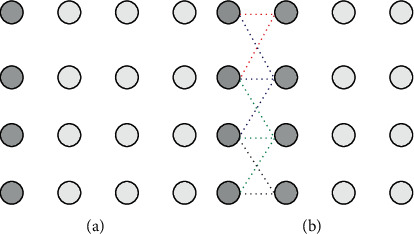
Illustration of the optimal splitting line search algorithm. Each gray filled circle represents its maximum cumulative energy value. (a) Initialization. (b) Expanding for the first time.

With the above given energy function, we define a splitting line as an optimal one if it gets the maximum average energy. In order to search for the optimal splitting line, we use dynamic programming associated with formula (17). The main steps of the searching algorithm can be summarized as follows :

  Step 1: initialization: as shown in Figure 6(a), each pixel in the first column is assigned with an energy value calculated using formula (17).
  Step 2: expanding sequentially until reaching the last column: for a pixel with (*x*, *y*) as coordinate, calculate its cumulative energy using (18). As Figure 6(b) indicates, each pixel in yth column can be expanded from three adjacent pixels in the previous column (y−1th column). At the same time, the index number of the coming pixel should be recorded using a certain structure, for example, using a matrix as (19):

(18) $$\begin{array}{c} E_{\text{cumulative}}\left(x,y\right)=\max\left\{E_{\text{cumulative}}\left.\left(i,y-1\right)\right|i=x-1,x,x+1\right\}+E\left(x,y\right), \end{array}$$

(19) $$\begin{array}{c} M_{\text{path}}\left(x,y\right)=\underset{i\in \left\{x-1,x,x+1\right\}}{\arg\max}E_{\text{cumulative}}\left(i,y-1\right). \end{array}$$

**Figure 7:**
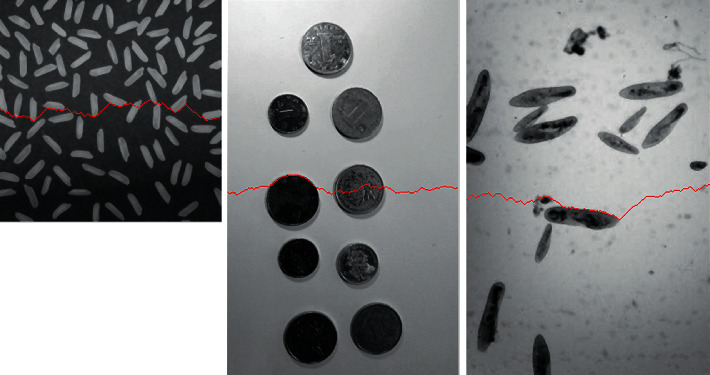
Optimal splitting lines of some uneven illumination images.

  Step 3: find the maximum cumulative energy in pixels of the last column and backtrack the optimal split line using matrix *M*_path_ constructed in Step 2.
  An uneven illumination image can be divided into two parts whose illumination is more consistent using the above introduced optimal splitting line search method.  Figure 7 demonstrates the optimal splitting lines acquired by the proposed algorithm on some testing uneven illumination images. It is obvious that illumination in the two parts of each image divided by the splitting line is more consistent, and the optimal splitting line tends to avoid crossing the foreground objects as designed.

### 3.4. Partition Technology-Based Thresholding Methods

After implementing the partition step, we can split the processed image I into two parts denoted as Ip1 and Ip2. In this section, we will discuss automatic thresholding segmentation for uneven illumination images based on the two split parts, Ip1 and Ip2. There are two typical strategies, which are considering Ip1 and Ip2 separately or computing a uniform threshold for both Ip1 and Ip2. Consequently, we propose two schemes for uneven illumination image thresholding based on the partition technology. For scheme one, the improved 2D Otsu's method MAOTSU_2D is directly implemented on Ip1 and Ip2 separately, and we can obtain two threshold vectors for the corresponding two parts. On the other hand, we proposed a second scheme to calculate a uniform threshold vector. The main steps of the proposed second scheme are as follows:

  Step 1: generate 2D histogram using the improved constructing strategy proposed in Section 3.1.
  Step 2: calculate the between-class variance *tr*(*σ*_*B*_^*p*1^), *tr*(*σ*_*B*_^*p*2^) for Ip1 and Ip2, respectively, using formula (7) for each threshold pair (*s*, *t*) in threshold value space Φ={(*s*, *t*)|0 < *s*, *t* < *L*}.
  Step 3: compute the optimal threshold pair (*s*^*∗*^, *t*^*∗*^) by solving the following optimization problem:

(20) $$\begin{array}{c} \left(s^{*},t^{*}\right)=\underset{0<s<L,0<t<L}{\arg\max}\left(tr\left(\sigma_{B}^{p1}\right)\times tr\left(\sigma_{B}^{p2}\right)\right) \end{array}$$

  The object function of the proposed scheme 2 includes two terms, *tr*(*σ*_*B*_^*p*1^) and *tr*(*σ*_*B*_^*p*2^), and they are multiplied together to form the whole between-class variance metric in order to maximize the between-class variance of both Ip1 and Ip2. In this paper, we calculate the optimal threshold pair (*s*^*∗*^, *t*^*∗*^) by searching the whole threshold value space Φ.

## 4. Experimental Results and Analysis

In order to verify the effectiveness of the proposed methods, we conducted experiments on a personal computer with Intel Core 2.30 GHz CPU and 4.0 GB memory. Original 2D Otsu's method, MAOTSU_2D without postprocessing, and MAOTSU_2D, as well as latest two 1D Otsu's methods (Cao's method and DVE) were selected to compare with our proposed schemes. All algorithms are implemented using Matlab R2012b, and experiments are conducted for verifying algorithms' robustness to salt-and-pepper noise (Section 4.1) and binarization ability on uneven illumination images (Section 4.2) and on regular cell images (Section 4.3). All algorithms are evaluated both quantitatively and qualitatively.

For quantitative testing, misclassification error (ME) and dice similarity coefficient (DSC) are adopted as the evaluation metrics. The definition of ME is described as follows:

(21) $$\begin{array}{c} \text{ME}=1-\frac{\left|F_{o}\cap F_{T}\right|+\left|B_{o}\cap B_{T}\right|}{\left|B_{T}\right|+\left|F_{T}\right|}, \end{array}$$

where *F*_*o*_ and *B*_*o*_ represent pixel sets of foreground and background and *F*_*T*_ and *B*_*T*_ are manually labelled foreground and background, respectively. In the rest of this section, we will separately evaluate the algorithm's robustness to salt-and-pepper noise and segmentation ability on uneven illumination images.

The other evaluation metric DSC can be expressed as the following formula:

(22) $$\begin{array}{c} \text{DSC}=\frac{2\text{TP}}{2\text{TP}+\text{FP}+\text{FN}}, \end{array}$$

where TP, FP, and FN are true positive, false positive, and false negative, respectively. In this paper, TP represents the total number of correctly detected foreground pixels, and FP and FN are the number of incorrect foreground pixels and miss-detected foreground pixels, respectively.

### 4.1. Testing of Algorithms' Robustness to Salt-and-Pepper Noise

**Figure 8:**
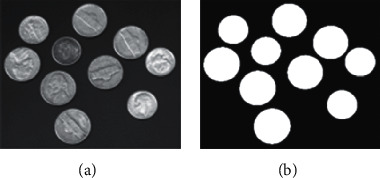
Testing image coins and corresponding ground truth. (a) Image coins. (b) Manually labelled ground truth.

In this section, we experimentally discuss the robustness of the proposed methods to salt-and-pepper noise. The testing image is the widely used image coins, and we had manually labelled the binary segmentation result as the ground truth as shown in Figure 8.

**Figure 9:**
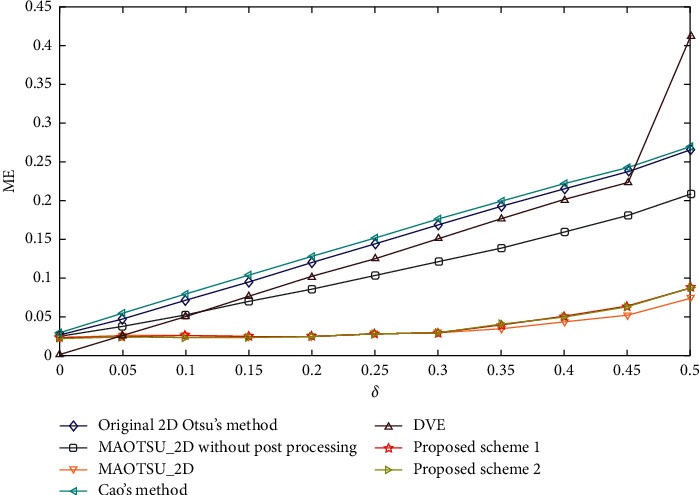
Relationship between ME and value of noise intensity parameter *δ*.

Figure 9 demonstrates the relationship between ME and noise intensity parameter *δ* which is ranging from 0 to 0.5. It is obvious that the two proposed schemes are more robust compared with original 2D Otsu's method, MAOTSU_2D without postprocessing, and 1D Otsu's methods including Cao's method and DVE algorithm, whose ME values are growing rapidly with increasing noise. Especially when the noise intensity parameter *δ* increases from 0 to 0.3, the growth of the corresponding ME values of the two proposed schemes is almost negligible. MAOTSU method showed similar relationship between ME and *δ* as the proposed schemes. However, as we discussed previously, while 2D histogram of original 2D Otsu's method is constructed using original image and average image, median-average image instead of average image is adopted in MAOTSU_2D. In fact, the rapid change of ME of MAOTSU_2D without postprocessing indicates that the robustness of MAOTSU_2D to salt-and-pepper noise to a large extent depends on the postprocessing.

**Figure 10:**
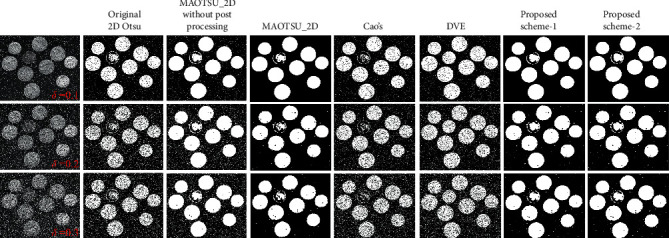
Segmentation results of each algorithm on salt-and-pepper corrupted images.

Figure 10 shows the segmentation results of each algorithm on images with different noise intensity. The two proposed schemes using the modified 2D histogram perform better than original 2D Otsu's method, MAOTSU_2D without postprocessing, and Cao's method. While DVE method can better segment foreground objects, it fails to deal with noises. The comparison results also indicate the effectiveness of the improved 2D histogram construction strategy. In addition, the proposed methods are more robust in foreground area compared to MAOTSU_2D with the postprocessing technology. Although it is remarkable that MAOTSU_2D method can better process noise pixels in background region, our proposed schemes are still competitive.

### 4.2. Evaluation of Segmentation Ability on Uneven Illumination Images

**Figure 11:**
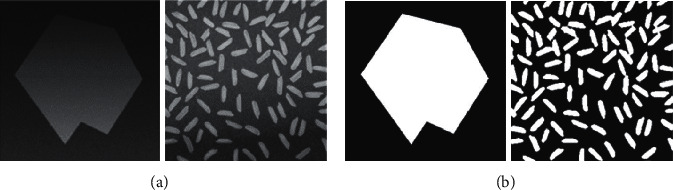
Synthetic and real world testing images and their corresponding ground truth. (a) Original images. (b) Corresponding manually labelled ground truth.

In this section, experiments are conducted to verify the effectiveness of our proposed schemes on uneven illumination images. We test different methods on both synthetic and real world images as shown in Figure 11 with manually labelled ground truth.

**Figure 12:**
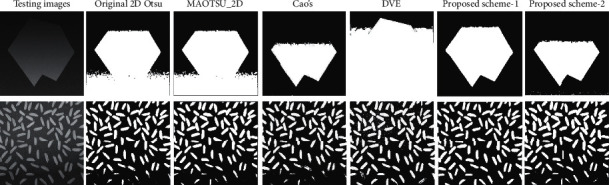
Segmentation results of each algorithm on the synthetic and real world images.

Table 1 is the quantitative results of all testing methods on synthetic image and real world image rice. The ME values of the proposed schemes are smaller than those of the original 2D Otsu's method, MAOTSU_2D, and 1D Otsu's methods on the synthetic image and the DSC values of the proposed schemes are larger than the compared methods. The improvement is more significant on the synthetic image for proposed scheme 1, showing lowest ME and highest DSC. For real world image rice, although the improvements of ME and DSC seem not very remarkable, the proposed methods can significantly augment the detection rate of the foreground objects in the bottom region shown in Figure 12.

Figure 12 exhibits the segmentation results of all the testing methods. The weakness of original 2D Otsu's method, MAOTSU_2D, and DVE algorithm can be found obviously from the segmentation results on the synthetic image. While the compared methods extensively misclassify the background as foreground at the bottom region, the two proposed schemes can better detect the real foreground object. At the same time, our proposed methods perform better on real world image rice. On one hand, the proposed scheme-1 can detect almost all the foreground objects; on the other hand, the proposed scheme-2 misses less foreground objects than the original 2D Otsu's method, MAOTSU_2D, and Cao's method. Although DVE can detect most foreground objects, the result images contain a lot of fake foreground pixels at the top region. Collectively these experimental results verify the effectiveness of the proposed schemes on the synthetic and real world images.

**Figure 13:**
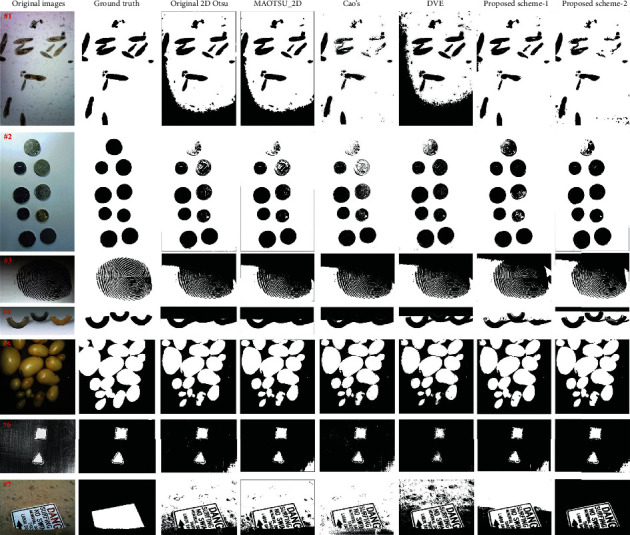
Segmentation results of each algorithm on other uneven illumination images.

For further verification, we test our method on some other real world uneven illumination images shown in Figure 13. The manually labelled ground truth for each image is shown in the second column. The comparison results demonstrate the advantage of our proposed methods. For example, the binarization results of the proposed method for images #1, #2, #3, #4, and #7 are much closer to the corresponding ground truth compared with those of other algorithms. For image #5, the results are similar qualitatively among all of the testing methods except DVE. For image #6, DVE's output is closer to the ground truth, but the results of other compared algorithms are also competitive.

Next, we evaluate the effectiveness of all testing methods quantitatively. Tables 2 and 3 are the ME and DSC values acquired for each of the algorithms on the seven real world testing images. The proposed method could reach best ME and DSC values for all images except for image #6, indicating best segmentation results. On average, our proposed method lowers the ME value by almost 15% and increases the DSC value by 10%.

### 4.3. Evaluation of Segmentation Ability on a Cell Dataset

In order to evaluate the performance of the proposed methods on other real world images, in this section, all the compared algorithms are tested on a cell dataset, which was introduced by Xing et al. in [14]. The cell dataset contains 22 cell images and the corresponding ground truths are manually labelled.

**Figure 14:**
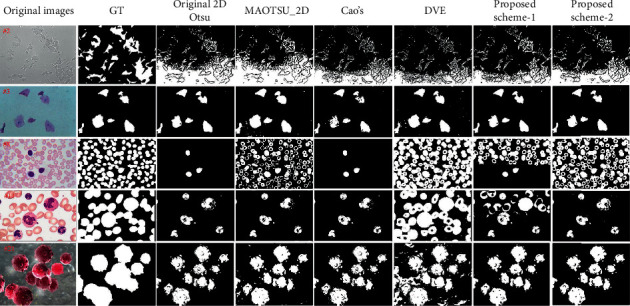
Segmentation results of each algorithm on cell images.

Figure 14 shows part of the segmentation results of the tested algorithms. All methods get similar segmentation results on image #3. The proposed schemes can produce more competitive results on images #2, #8, and #13 than other methods except DVE. For image #21, DVE achieves worst segmentation result while all other methods could generate similar results.

The average ME and DSC values of each algorithm on all 22 cell images can be found in Table 4. The performance of our proposed method is better than that of original 2D Otsu, MAOTSU_2D, and Cao's method by achieving lower ME and higher DSC values and is comparable to that of DVE algorithm. The proposed method could achieve lowest average ME value while DVE could acquire highest average DSC value. These quantitative results prove that the proposed method not only is effective on uneven illumination images, but can also achieve competitive results on regular illumination real world images.

### 4.4. Discussion

In the previous experimental sections, robustness and segmentation performance of the improved 2D Otsu's method are tested. The improved method can deal with salt-and-pepper noise properly compared to original 2D and 1D Otsu's methods and can make a significant improvement on segmenting uneven illumination images both qualitatively and quantitatively. At the same time, for normal cell images segmentation, the performance of the proposed method is still quite competitive. However, as a global thresholding method, there exists a performance limit, which means that, for some uneven images, no global threshold could be found that can effectively extract foreground. Moreover, the foreground detection ability of the proposed method on the cell dataset is slightly weaker than our previous DVE method which can be found partially in Figure 14. The smaller average DSC value may also indicate its lower foreground detection ability to some extent.

## 5. Conclusion

To alleviate the limitations of 2D Otsu's method to salt-and-pepper noise and uneven illumination for image segmentation, we have proposed a robust 2D Otsu's method in this paper. A 2D histogram construction strategy based on median and average filters is introduced, and an energy based image partition method is developed for uneven illumination image segmentation. Experiments are conducted on both synthetic and real world images to evaluate the performance of the proposed method. The qualitative and quantitative evaluations collectively indicate that the proposed method is more robust to salt-and-pepper noise and performs better on uneven illumination images compared with original Otsu's method, MAOTSU_2D, Cao's method, and DVE, demonstrating its better flexibility in automatic image thresholding.

## Figures and Tables

**Table 1:** ME and DSC values of each algorithm on synthetic and real world images.

	ME and DSC	
	Synthetic image	Real world image (rice)
Original 2D Otsu	0.2806, 0.7097	0.0524, 0.9074
MAOTSU_2D	0.2774, 0.7121	0.0527, 0.9077
Cao's	0.1397, 0.7839	0.0686, 0.8750
DVE	0.4359, 0.6425	**0.0399, 0.9330**
Proposed scheme 1	**0.0284, 0.9625**	0.0425, 0.9267
Proposed scheme 2	0.1232, 0.8154	0.0512, 0.9141

Bold values represent the best evaluation metric values among all the compared algorithms.

**Table 2:** ME values of each algorithm on real world uneven illumination images.

Image	Original 2D Otsu	MAOTSU_2D	Cao's	DVE	Proposed scheme 1	Proposed scheme 2
#1	0.0404	0.0418	0.0413	0.2945	**0.0269**	0.0298
#2	0.2056	0.2058	0.0596	0.0303	**0.0233**	0.0370
#3	0.3512	0.3618	0.3659	0.3274	0.2745	**0.2690**
#4	0.3995	0.4053	0.4137	0.3336	**0.0816**	0.1790
#5	0.0808	0.0798	0.0893	0.1125	0.0774	**0.0699**
#6	0.0349	0.0339	0.0352	**0.0229**	0.0380	0.0336
#7	0.6754	0.6808	0.7771	0.5149	0.5970	**0.1168**
Avg.	0.2554	0.2585	0.2546	0.2337	0.1598	**0.1050**

Bold values represent the best ME values among all the compared algorithms.

**Table 3:** DSC values of each algorithm on real world uneven illumination images.

Image	Original 2D Otsu	MAOTSU_2D	Cao's	DVE	Proposed scheme 1	Proposed scheme 2
#1	0.9755	0.9747	0.9773	0.8032	**0.9836**	0.9817
#2	0.8710	0.8709	0.9645	0.9815	**0.9870**	0.9796
#3	0.6857	0.6732	0.6682	0.7132	0.7701	**0.7759**
#4	0.6753	0.6688	0.6604	0.774	**0.9505**	0.8789
#5	0.9170	0.9180	0.9074	0.8803	0.9207	**0.9290**
#6	0.7306	0.7338	0.7265	**0.7537**	0.7100	0.7361
#7	0.3207	0.3162	0.2964	0.3741	0.3408	**0.7015**
Avg.	0.7394	0.7365	0.7430	0.7543	0.8090	**0.8547**

Bold values represent the best DSC values among all the compared algorithms.

**Table 4:** Average ME and DSC values of each algorithm on cell images.

Metric	Original 2D Otsu	MAOTSU_2D	Cao's	DVE	Proposed scheme 1	Proposed scheme 2
ME	0.1606	0.1492	0.1639	0.1457	0.1374	**0.1371**
DSC	0.7302	0.7709	0.7148	**0.7996**	0.7831	0.7803

Bold values represent the best ME and DSC values on cell images among all the compared algorithms.

## Data Availability

The data used to support the findings of this study are available from the corresponding author upon request.
